# Audiovestibular Dysfunction Related to Anti-Phospholipid Syndrome: A Systematic Review

**DOI:** 10.3390/diagnostics14222522

**Published:** 2024-11-11

**Authors:** Jiann-Jy Chen, Chih-Wei Hsu, Yen-Wen Chen, Tien-Yu Chen, Bing-Yan Zeng, Ping-Tao Tseng

**Affiliations:** 1 Prospect Clinic for Otorhinolaryngology & Neurology, Kaohsiung City 81166, Taiwan; jiannjy@yahoo.com.tw (J.-J.C.); kevinachen0527@gmail.com (Y.-W.C.); 2 Department of Otorhinolaryngology, E-Da Cancer Hospital, I-Shou University, Kaohsiung 80756, Taiwan; 3 Department of Psychiatry, Kaohsiung Chang Gung Memorial Hospital and Chang Gung University College of Medicine, Kaohsiung 83340, Taiwan; harwicacademia@gmail.com; 4 Department of Psychiatry, Tri-Service General Hospital, School of Medicine, National Defense Medical Center, Taipei 11490, Taiwan; verducciwol@gmail.com; 5 Institute of Brain Science, National Yang Ming Chiao Tung University, Taipei 112, Taiwan; 6 Institute of Biomedical Sciences, National Sun Yat-sen University, Kaohsiung 80424, Taiwan; 7 Department of Internal Medicine, E-Da Dachang Hospital, I-Shou University, Kaohsiung 80706, Taiwan; 8 Department of Psychology, College of Medical and Health Science, Asia University, Taichung 413305, Taiwan; 9 Institute of Precision Medicine, National Sun Yat-sen University, Kaohsiung 804201, Taiwan

**Keywords:** anti-phospholipid syndrome, cochleopathy, vestibular, sensorineural hearing loss, treatment

## Abstract

***Background:*** Anti-phospholipid syndrome (APS) has emerged as a significant issue in autoimmune diseases over recent decades. Its hallmark feature is thromboembolic events, potentially affecting any vascularized area including the microcirculation of the inner ear. Since the first case report of APS-related audiovestibular dysfunction described in 1993, numerous reports have explored the association between APS-related antibodies and audiovestibular dysfunction. These studies indicate a higher prevalence of APS-related antibodies in patients with sensorineural hearing loss compared to healthy controls. Unlike other idiopathic hearing loss disorders, audiovestibular dysfunction associated with APS may respond to appropriate treatments, highlighting the importance of timely recognition by clinicians to potentially achieve favorable outcomes. Therefore, this systematic review aims to consolidate current evidence on the characteristics, pathophysiology, assessment, and management of audiovestibular dysfunction linked to APS. ***Methods:*** This systematic review utilized electronic searches of the PubMed, Embase, ClinicalKey, Web of Science, and ScienceDirect online platforms. The initial search was performed on 27 January 2024, with the final update search completed on 20 June 2024. ***Results:*** Based on theoretical pathophysiology, anticoagulation emerges as a pivotal treatment strategy. Additionally, drawing from our preliminary data, we propose a modified protocol combining anticoagulants, steroids, and non-invasive brain stimulation to offer clinicians a novel therapeutic approach for managing these symptoms. ***Conclusions:*** Clinicians are encouraged to remain vigilant about the possibility of APS and its complex audiovestibular manifestations, as prompt intervention could stabilize audiovestibular function effectively.

## 1. Introduction

There has been a long-standing debate regarding the association between audiovestibular dysfunction and autoimmune diseases [1]. Several reports have explored this link in specific autoimmune conditions such as systemic lupus erythematosus [2,3], rheumatoid arthritis [4,5], and autoimmune thyroiditis [6]. However, while some of these conditions have clear physiological evidence supporting their association [3,7,8], others lack satisfactory pathophysiological explanations connecting autoimmune diseases with dysfunction of the audiovestibular system [6]. For instance, only a few diseases have histopathological evidence establishing a direct relationship between autoimmune reactions and the audiovestibular system, such as systemic lupus erythematosus [2]. According to our previous reports, a well-recognized physiopathology of immune-mediated audiovestibular dysfunction would lead to a relatively satisfactory treatment result [3,9,10].

Anti-phospholipid syndrome (APS) is characterized by recurrent thromboembolic events and an inflammatory process [11]. It can sometimes occur alongside other autoimmune diseases, such as systemic lupus erythematosus (present in around one third of cases) [12]. Depending on its association with other autoimmune diseases, APS is classified as “primary” (APS alone) or “secondary” (APS with other autoimmune diseases) [12,13]. Circulating anti-phospholipid antibodies bind to phospholipids and phospholipid-bound proteins on cell membranes, potentially activating endothelial cells, platelets, and leukocytes, thereby promoting thrombus formation and subsequent autoimmune and inflammatory reactions [14,15].

To diagnose APS [16], patients must meet at least one clinical criterion and one laboratory criterion. Clinical criteria include evidence of (1) vascular thrombosis or (2) pregnancy morbidity. Laboratory criteria consist of (1) lupus anticoagulants, (2) anti-cardiolipin antibodies, or (3) anti-β2-glycoprotein I antibodies. Moreover, to meet laboratory criteria, patients must test positive for one or more of these antibodies on at least two occasions separated by at least 12 weeks [16].

Since the first case report by Hisashi and colleagues [17], the role of APS in the pathophysiology of audiovestibular dysfunction has become a significant topic in otolaryngology research [18,19,20,21,22,23]. Before serologically proven, the APS-related audiovestibular dysfunction might mimic the presentation of Meniere disease or idiopathic sensorineural hearing loss [24]. As addressed earlier, macro- or micro-thromboembolism is a key feature of APS [25]. Microcirculation plays a crucial role in maintaining inner ear homeostasis [26]. Therefore, any disruption or occlusion of inner ear microcirculation theoretically leads to subsequent audiovestibular dysfunction. Although not always, it might present as sudden sensorineural hearing loss when the occlusion of microcirculation occurs abruptly [27]. Consequently, there has been an increasing number of reports investigating the association between audiovestibular dysfunction and APS-related antibodies [24,27,28,29,30,31,32,33,34,35,36,37,38]. These studies have demonstrated a higher prevalence of elevated APS-related antibodies in patients with sensorineural hearing loss compared to healthy controls [29,39]. Additionally, a large-scale study examining the risk of sudden sensorineural hearing loss across various autoimmune diseases found that patients with APS had a significantly higher risk compared to those without autoimmune diseases [37]. However, this finding has not been consistently replicated in other studies [40,41,42,43]. Audiovestibular dysfunction related to APS can manifest as either sudden-onset or progressive [11].

Unlike other idiopathic hearing loss disorders, audiovestibular dysfunction associated with APS may respond to appropriate treatments [18,19]. Therefore, timely recognition of this condition could enable clinicians to promptly identify and manage audiovestibular dysfunction related to APS, potentially improving clinical outcomes. Hence, the objective of this systematic review is to summarize the current evidence on the characteristics, pathophysiology, assessment, and treatment of audiovestibular dysfunction associated with APS.

## 2. Methods and Materials

**Figure 1:**
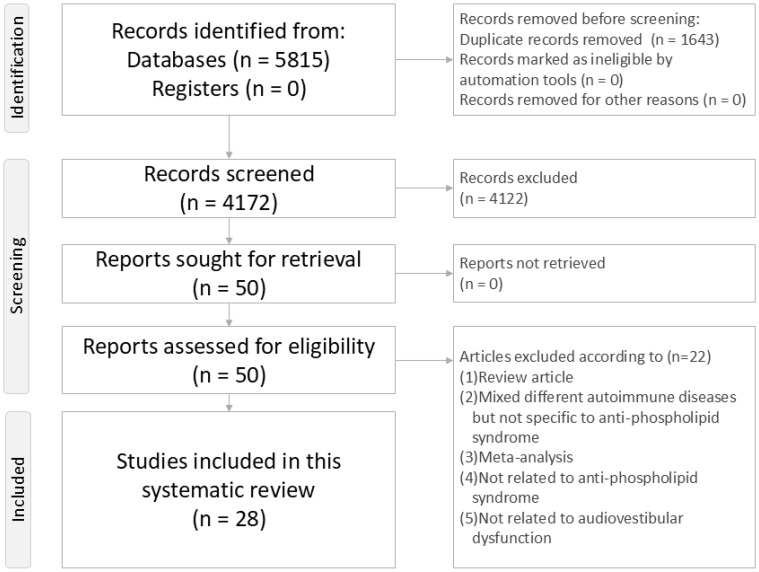
Flowchart of the whole systematic review procedure.

This systematic review adheres to the Preferred Reporting Items for Systematic Reviews and Meta-Analyses (PRISMA) statement (Table S1 and Figure 1) [44]. The review protocol was registered on the INPLASY platform (INPLASY202460076, https://inplasy.com/inplasy-2024-6-0076/, accessed on 21 October 2024).

### 2.1. Literature Search Strategy

The systematic review utilized electronic searches of the PubMed, Embase, ClinicalKey, Web of Science, and ScienceDirect online platforms. Detailed search strategies and keywords for each platform are provided in Table S2. Additionally, a manual search of reference lists from included articles was conducted. The initial search was performed on 27 January 2024 with the final update search completed on 20 June 2024. If insufficient data were available in the original papers, corresponding authors were contacted via email to request additional information.

### 2.2. Inclusion and Exclusion Criteria

This systematic review focused on audiovestibular issues related to APS, including characteristics, pathophysiology, examination, and treatment. Inclusion criteria were as follows: (a) studies examining audiovestibular issues related to APS; (b) case reports/series, observational trials, case–control trials, or randomized controlled trials; and (c) studies including patients with APS.

Exclusion criteria were as follows: (a) studies not including patients with APS; (b) studies not addressing characteristics, pathophysiology, examination, or treatment related to audiovestibular dysfunction in APS; and (c) animal studies. Review articles were used for manual extraction of relevant articles from their reference lists. The main reasons for the exclusion of 4122 records at the initial screening stage of “Title–-Abstract Screening” included the studies not being related to APS, not being related to audiovestibular dysfunction, and not being human trials. After passing the initial screening stage, the remaining 50 records entered full-text screening. Among them, 22 records were excluded and the reasons are detailed in Table S3.

### 2.3. Article Screening Process

Following the electronic searches across all five databases and the application of the inclusion/exclusion criteria based on titles and abstracts, eligible articles were subjected to full-text examination. Duplicate articles were manually removed, and the remaining articles underwent full-text screening to determine final inclusion.

### 2.4. Data Extraction

Data extraction was performed by Ping-Tao Tseng, who conducted full-text examinations to extract data on characteristics, pathophysiology, examination, and treatment related to APS.

### 2.5. Article Quality Grading

Clinical studies were assessed for quality using the Newcastle–Ottawa Scale by Jiann-Jy Chen and Ping-Tao Tseng [45] (Table S4).

## 3. Summary of the Currently Available Evidence

### 3.1. Characteristics

The core pathophysiology of APS hinges on its thromboembolic effects, affecting arteries or veins in both the central nervous system and peripheral organs [21]. APS can manifest as primary (without concurrent autoimmune diseases) or secondary (often associated with diseases like systemic lupus erythematosus) [21]. Although anti-phospholipid antibodies may be a non-specific response to infections rather than an independent autoimmune disease [46], they are central to the syndrome’s definition rather than termed as anti-phospholipid disease.

APS’s primary features stem from multifocal thrombosis. Unlike other autoimmune conditions such as systemic lupus erythematosus, histological examinations of blood clots typically reveal organized thrombi with platelet aggregation and fibrin, often lacking significant inflammation or vasculitis [27]. Neuroimaging reports show findings like severe stenosis, long-segment vessel wall thickening, and homogeneous vessel wall enhancement in patients with APS [47]. While anti-cardiolipin antibodies may coexist with other autoimmune diseases like mixed connective tissue disorders [48], their presence more strongly correlates with sensorineural hearing loss than with these mixed connective tissue disorders [1].

Audiovestibular dysfunction associated with APS can present unilaterally or bilaterally [49], with a variable course ranging from sudden onset to chronic progression [20,23,31,36]. Rarely, sudden sensorineural hearing loss may spontaneously recover [50]. Often, patients initially present with other APS symptoms before later developing sensorineural hearing loss. Audiovestibular dysfunction as the initial presentation of APS is relatively uncommon [23,51], with the vestibular system less frequently affected compared to the auditory system [52]. Vestibular dysfunction in APS may result from impairment to vestibulo-cerebellar structures or vestibular nuclei [53]. Studies on sensorineural hearing loss suggest significant impairment across various frequencies, particularly in middle to low frequencies, compared to standard levels [54].

### 3.2. Epidemiology

Increased levels of anti-phospholipid antibodies and anti-HSP-70 antibodies have been noted in idiopathic sudden sensorineural hearing loss, with prevalence rates of 33.3% and 25.4%, respectively [29], higher than those in healthy controls (9.4% [39] and 4.2% [55]). The prevalence of serologically positive anti-cardiolipin antibodies in sudden or progressive sensorineural hearing loss ranges from 0.7% to 27% [35,56]. Similar prevalence rates are found in patients with Meniere’s disease, where 27% of unilateral and 12% of bilateral cases show elevated anti-phospholipid antibodies (population norm 6–9%) [21]. Coexisting antinuclear antibodies are frequently found, with prevalence rates ranging from 17% to 43% [34,57,58], reflecting the interconnected etiology of autoimmune diseases. A recent study reported that 49% of patients with sudden hearing loss and 50% with progressive hearing loss tested positive for anti-phospholipid antibodies [59].

### 3.3. Physiopathology

**Figure 2:**
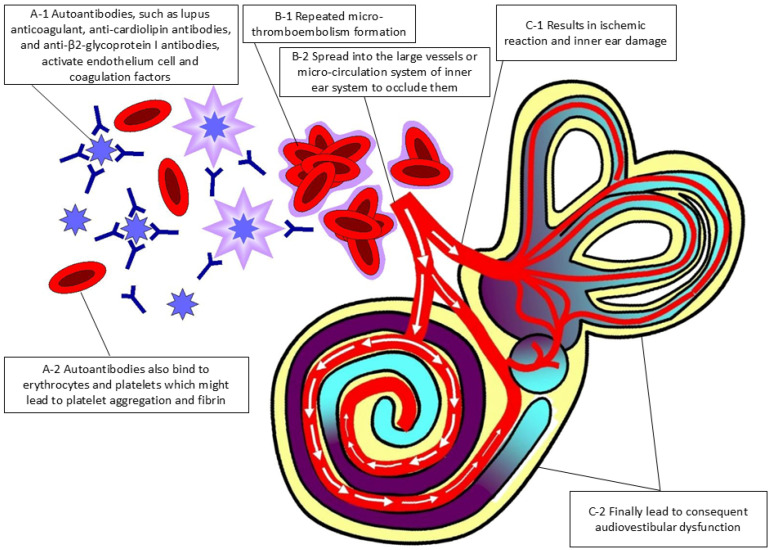
Schematic diagram of the physiopathology of anti-phospholipid syndrome in audiovestibular dysfunction.

The overall pathophysiological reaction involving APS-related antibodies and the formation of thromboembolism, which ultimately contributed to patients’ audiovestibular dysfunction, is illustrated in Figure 2, which was drawn by the first author.

#### 3.3.1. Lupus Anticoagulant

Lupus anticoagulant, often accompanied by anti-cardiolipin antibodies, plays a crucial role in the prothrombotic effects of APS [60,61]. It is considered a stronger risk factor for thrombosis compared to anti-cardiolipin antibodies [62]. In a large-scale case–control study of autoimmune antibodies in idiopathic sudden sensorineural hearing loss, lupus anticoagulant prevalence was significantly higher in patients than in healthy controls (8.4% versus 0.6%) [32]. Patients with lupus anticoagulant are more prone to thrombotic events [62], whereas those primarily with anti-cardiolipin antibodies tend to exhibit more severe disease manifestations [62]. Further, recurrent thrombotic events typically occur in the same vascular bed [63].

#### 3.3.2. Anti-Cardiolipin Antibodies

Research indicates that positivity for anti-cardiolipin antibodies is an independent risk factor for sensorineural hearing loss [32]. Another study linking elevated anti-phosphatidylserine antibodies (a subtype of anti-phospholipid antibodies) with normal-tension glaucoma also associates them with hearing loss [31]. The coexistence of both anti-phosphatidylserine IgM and IgG antibodies suggested an active (IgM) and persistent (IgG) autoimmune process in such patients [31]. These antibodies primarily target negatively charged phospholipids such as cardiolipin and β2-glycoprotein I on tissue surfaces [29]. For example, the cardiolipin resided in the inner mitochondrial membrane [64]. The production of the anti-phospholipid antibodies could be invoked by exposure to viral infection [65,66], such as human immunodeficiency virus and hepatitis C virus [67]. The hypothetic linkage between viral infection and activation of anti-phospholipid antibodies mainly relies on two hypotheses: (1) the viral sequence might mimic the phospholipid-binding site or host self-sequence [29] and (2) the cardiolipin located at the mitochondrial membrane might degrade and release into extracellular area as a result of oxidative stress and infection [68]. Though controversial, these antibodies likely interfere with platelet surface homeostasis or endothelial cell function, contributing to vaso-occlusive manifestations [29]. Microvascular obstruction has been proposed as a link between audiovestibular dysfunction and systemic autoimmune diseases, such as systemic lupus erythematosus [2] or APS [29]. It might be related to (a) the humoral-type antibody [69] and cell-mediated cytotoxicity [70] targeting over the inner ear antigen, (b) the immune complex deposition in the microcirculation of the inner ear [26], or (c) the occlusion in the microcirculation of the internal ear by micro-thrombosis formation [17,19] related to anti-phospholipid antibodies [71]. A high prevalence up to 50% of intra-cranial arterial thrombosis, in either the micro-vascular system or large vessels, and its consequent neuropsychiatric sequalae had been found in patients with APS [72], which could serve as a linkage between APS and an impaired audiovestibular system circulation system. Vessels of any size may be targeted and, in one study, none of the vessels could be spared [73]. To be specific, the anti-phospholipid antibodies could activate cochlear vessel endothelial cells [74], either via direct effect or via the stimulation of free radicals [75]. The aforementioned mechanism would lead to endothelial dysfunction and damage, and finally would result in local micro-thrombus formation and end-organ ischemia [74,75]. From another point of view, in the report by Green and colleagues, the authors concluded that the time association between the onset of sensorineural hearing loss and a subsequent additional thrombotic event and a lack of response to steroid treatment might suggest that the physiopathology pathway relies on a thrombotic pathway but not an inflammatory etiology [76]. Additionally, some rare cases, such as those with persistent stapedial artery, might be prone to be affected earlier by complications of APS due to anatomical variations in the circulation system [77].

However, most of the aforementioned evidence about the linkage between the anti-cardiolipin antibody reaction and audiovestibular dysfunction was derived from the interpretation of case reports and their response to immunosuppressive treatment [78]. Therefore, they were not direct evidence because there were no direct methods to determine cochlear blood flow [32]. On the other hand, researchers tried to find evidence through another aspect, the time association. To be specific, in recent clinical reports, researchers have demonstrated an association of the existence of anti-cardiolipin antibody with acute-phase idiopathic sudden sensorineural hearing loss [29]; however, the aforementioned anti-cardiolipin antibodies disappear 3 months later [34]. This time association between transiently elevated anti-cardiolipin antibodies (either related to viral infection or other causes) and sudden sensorineural hearing loss might explain the transient vascular damage and microthrombosis formation in such patients [34].

#### 3.3.3. Anti-β2-Glycoprotein I Antibodies

Reports suggest that anti-β2-glycoprotein antibodies may be associated with acute events like sudden sensorineural hearing loss, while other anti-phospholipid antibodies may correlate with progressive hearing loss [28]. A case report documented parietal lobe microvascular involvement in sudden sensorineural hearing loss associated with high anti-β2-glycoprotein and anti-cardiolipin antibody titers [20].

#### 3.3.4. Other Associated Autoantibodies

In addition to the aforementioned main autoantibodies, the heat shock protein (HSP) family, which is a set of evolutionarily conserved molecular chaperones [79], also played an important role in sensorineural hearing loss in patients with APS [29]. As we know, the HSP family serves as a protector for our tissues to resist various forms of stress including heat, ischemia, free radicals, and toxic agents [80]. However, because of its highly conserved properties during species’ evolutionary processes [79], it might be similar to HSPs in other infectious micro-organisms [81,82]. Therefore, it might become the source of cross-reaction between autoimmune activities and human HSPs [29]. HSP-70 was one of the HSP family members. HSP-70 in the inner ear system could be the target of immunologic reactions via the cellular immune response in the host during micro-organism infection, which would result in consequent cochlear dysfunction [83]. Further, sensorineural hearing loss accompanied with tinnitus would be more likely to be associated with positive anti-HSP-70 antibodies and anti-phospholipid antibodies than that without tinnitus [29]. However, despite the aforementioned physiopathological link between the HSP-70 immune reaction and cochlea dysfunction, only a few reports focused on serum anti-HSP-70 antibodies in patients with sensorineural hearing loss [29,83,84]. Among these reports, the prevalence of positive serum anti-HSP-70 antibodies in patients with sensorineural hearing loss was around 25.4–76.9% [29,83,84]. However, the application of anti-HSP-70 antibodies as a serologic marker in autoimmune disease-related sensorineural hearing loss remained controversial because of the following two concerns: First, available evidence regarding the detection of anti-HSP-70 antibodies in such patients is currently relatively lacking. Secondary, the anti-HSP-70 antibodies were also sometimes detected in healthy controls in low levels [29]; therefore, we should only take positive anti-HSP-70 antibodies into consideration if they are significantly increased in patients with sensorineural hearing loss.

### 3.4. Complication

Since APS is a systematic disease, its clinical presentation might vary widely in different organs or systems. To be specific, it could be either isolated to the audiovestibular system or widely disseminated to other organs. This depends on the sites of end organs involved with the physiopathology of thrombotic microangiopathy or thromboembolic events related to ischemia [24]. Further, the aforementioned multi-organ complications could be clinically silent and slow or rapidly progressive [24].

### 3.5. Diagnostic Tool

Given that anti-phospholipid antibodies primarily induce thrombotic processes in various vessels, assessing vessel health (thrombus formation, vessel wall integrity, blood flow velocity) may serve as an indicator of audiovestibular dysfunction related to APS [24]. Routine immunological laboratory tests, including antinuclear, antineutrophil cytoplasmic, anti-endothelial cell, anti-phospholipid/anti-cardiolipin, and anti-thyroid antibodies, are recommended when evaluating suspected immune-mediated inner ear disorders to enable early diagnosis [58].

### 3.6. Prognosis

Some researchers have attempted to classify APS into “primary” and “secondary” forms (the latter occurring alongside major autoimmune diseases like systemic lupus erythematosus). However, there appears to be little difference in the prognosis of audiovestibular dysfunction between these subtypes [24].

While several risk factors associated with the prognosis of sensorineural hearing loss have been identified, including the severity of hearing loss, the presence of vestibular symptoms, and patient age [85], only a few attempts have been made to predict the outcome of sensorineural hearing loss using biochemical parameters [29,86,87]. For instance, Gross et al. demonstrated a statistically significant association between anti-HSP-70 antibodies and the Siegel recovery grade in patients with sensorineural hearing loss [29]. Specifically, patients with positive anti-HSP-70 antibodies showed a significantly higher complete recovery rate compared to those without these antibodies [29]. This finding suggests that the presence of anti-HSP-70 antibodies indicates an autoimmune process in such patients, who are potentially responsive to immune modulation therapy such as steroid treatment [29]. Supporting this notion, Hirose et al. proposed that Western blot analysis for HSP-70 is the most effective test for predicting corticosteroid responsiveness, with a positive predictive value of 91% [30]. However, this predictive effect was not observed in another study by Suslu et al., where no association was found between a positive test for anti-HSP-70 antibody and response to corticosteroid treatment [33]. This discrepancy may be attributed to two factors: (1) the relatively small sample size in the study by Suslu et al. [33], and (2) differences in the tools used to detect anti-HSP-70 antibodies. Specifically, Suslu et al. reported only three cases with positive anti-HSP-70 antibodies [33] compared to eleven cases in the study by Hirose et al. [30]. Furthermore, in Suslu et al.’s study, the *p*-value for corticosteroid response in patients with anti-HSP-70 antibodies was 0.07, indicating proximity to statistical significance [33]. Additionally, while Western blotting showed positive predictive value in Hirose et al.’s study [30], ELISA did not yield significant predictive value in Suslu et al.’s study [33]. Although conclusive evidence regarding the choice of detection tool is lacking [33], Munari et al. reported that the ELISA method has a sensitivity of 84% and specificity of 93% for detecting anti-HSP-70 antibodies in the serum of such patients [88].

### 3.7. Treatment

#### 3.7.1. Anticoagulants

Currently, there are no definitive conclusions regarding the treatment of audiovestibular dysfunction related to APS. However, some researchers emphasize resolving the thromboembolic events associated with the syndrome. Specifically, regimens combining antithrombotic medications and immunotherapy are theoretically preferable for addressing the underlying physiopathology of APS [75]. Warfarin is identified as one potentially effective regimen for these patients [75].

Regarding the impact of anticoagulants on managing audiovestibular dysfunction in APS patients, there are differing viewpoints [24,89]. For instance, Wang et al. reported unsatisfactory outcomes with warfarin plus low-molecular-weight heparin, showing no improvement in hearing loss [23]. Similarly, Wiles et al. noted poor recovery from sudden sensorineural hearing loss despite continuous warfarin with or without aspirin [27]. In contrast, other reports suggest positive effects of anticoagulants. Cavallasca et al. demonstrated stabilization of hearing function without relapse after adding acenocoumarol therapy (INR 2-3) [19]. Similar stabilization and prevention of relapse were observed by Bir et al. [18], while Kang et al. linked the discontinuation of anticoagulants to the onset of sensorineural hearing loss, which reversed upon resuming therapy [90]. To be specific, in the report by Kang et al., a patient with primary APS continued with warfarin 5 mg/day and acetylsalicylic acid 100 mg/day for several years and had a sudden onset of tinnitus and hearing impairment few days after discontinuing the aforementioned anticoagulants. After resuming warfarin 5 mg/day and acetylsalicylic acid 100 mg/day, his audiometry revealed a recovery of hearing [90]. Vyse et al. illustrated the prophylactic benefits of long-term warfarin treatment (up to 5 years) in preventing recurrent thromboembolic events in these patients [91].

It has been noted that once inner ear thrombotic events occur, they tend to recur [63]. Therefore, some researchers recommend initiating long-term anticoagulant therapy, such as warfarin (target INR 3.0–4.0 for arterial prevention and 2.5–3.0 for venous prevention), to prevent future episodes of sensorineural hearing loss [27,92]. However, there are also reports opposing the long-term prescription of anticoagulants in otherwise healthy patients with APS [21].

#### 3.7.2. Steroids

Steroids have been widely used in various autoimmune inner ear diseases [93,94,95,96], exerting anti-inflammatory, immunosuppressive, and anti-edema effects [2]. This treatment can restore the capacity of otologic vessels by reducing inflammation and edema [2]. Additionally, the associated increase in systemic blood pressure from steroid use may enhance blood perfusion in the auditory artery [2]. Moreover, steroid application may protect neural tissues from ischemic injury, stabilize vascular endothelium, and restore the blood–brain barrier [97]. High-dose steroid therapy has shown beneficial effects in restoring hearing function in patients with APS, as reported by Bir et al., where hearing was recovered after a regimen of prednisolone (80 mg/day initially, tapering over 3 months) [18]. Similarly, in the study by Hisashi et al., combining steroid therapy with prostaglandin E1 or ticlopidine contributed to hearing function restoration in patients with anti-cardiolipin antibodies [98]. However, some studies do not universally support the beneficial effects of steroids in patients with positive anti-phospholipid antibodies [22,76,99]. For instance, in the report by Hisashi et al., hearing loss showed only partial improvement after steroid treatment without additional anticoagulants [17].

#### 3.7.3. Diuretics

Hydrochlorothiazide, with or without glycerol, can alleviate inflammation-induced tissue edema through its diuretic and dehydrating effects [100]. The rationale for using diuretics to alleviate sensorineural hearing loss in autoimmune diseases stems partly from steroids’ ability to reduce edema in autoimmune inner ear diseases [2], thereby potentially restoring otologic vessel capacity [2]. However, diuretic use in sensorineural hearing loss related to APS remains rare due to concerns about exacerbating ischemia in the inner ear microcirculation. Compadretti et al. reported the use of hydrochlorothiazide with glycerol to restore hearing function in a case of APS [101].

#### 3.7.4. Plasmapheresis

Plasmapheresis, or plasma exchange, has been used to manage audiovestibular dysfunction in patients with systemic lupus erythematosus [102,103]. While direct studies on the efficacy of plasmapheresis for managing audiovestibular dysfunction in patients with APS are lacking, there is one case report demonstrating a correlation between decreased anti-phospholipid antibody titers and improved hearing function following plasmapheresis in a systemic lupus erythematosus patient [104].

#### 3.7.5. Modified Anticoagulant, Steroid, and Non-Invasive Brain Stimulation Protocol for Managing Audiovestibular Dysfunction Related to APS

**Figure 3:**
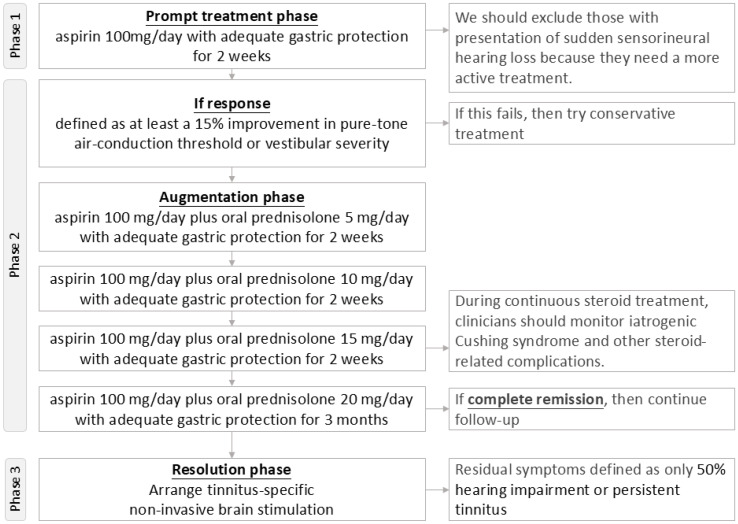
Flowchart of the modified anticoagulants plus steroid and non-invasive brain stimulation treatment protocol to manage anti-phospholipid syndrome-related audiovestibular dysfunction. Note: this is a proposal of a future study protocol.

In our preliminary research, we developed a modified treatment protocol combining anticoagulants, steroids, and non-invasive brain stimulation (Figure 3) specifically for managing audiovestibular dysfunction associated with APS. This protocol, based on Alexander’s approach for various autoimmune inner ear diseases, is structured as a three-phase trial [93].

Phase 1 (Prompt Treatment Phase) involves administering aspirin 100 mg/day for 2 weeks to evaluate patients’ response to anticoagulant therapy. A positive response is defined as at least a 15% improvement in pure-tone air-conduction threshold or reduction in vestibular severity.

Phase 2 (Augmentation Phase) targets patients who respond to anticoagulant treatment. They continue aspirin 100 mg/day throughout the treatment period and additionally receive oral prednisolone starting at 5 mg/day for 2 weeks, gradually increasing by 5 mg/day every 2 weeks until reaching a maintenance dosage of prednisolone 20 mg/day. The final dosage is maintained for 3 months.

Phase 3 (Resolution Phase) addresses patients with residual symptoms such as 50% hearing impairment or persistent tinnitus, who undergo non-invasive brain stimulation specifically targeted at tinnitus management [105].

### 3.8. Limitation

There are several limitations to address in this study, including a lack of direct pathological evidence to prove that APS induces audiovestibular dysfunction, the lack of available full texts for some articles, and limited evidence regarding the proposed treatment protocol.

## 4. Conclusions

This systematic review has synthesized current knowledge on audiovestibular dysfunction associated with APS. Unlike other autoimmune diseases, the predominant features of audiovestibular dysfunction in these patients stem largely from specific multiple thromboembolic events affecting either a single organ or multiple organs. Consequently, the most relevant and effective treatment strategy focuses on the use of anticoagulants, despite debates surrounding their long-term prescription. Given that APS presents as a syndrome rather than a distinct disease, its manifestations can vary widely and often coexist with other autoimmune conditions. Therefore, clinicians should remain vigilant for potential cases of APS and its complex audiovestibular dysfunction, as timely intervention may aid in stabilizing audiovestibular function, though complete recovery may not always be achievable.

## Supplementary Materials

The following supporting information can be downloaded at: https://www.mdpi.com/article/10.3390/diagnostics14222522/s1, Table S1: PRISMA 2020 checklist of current systematic review; Table S2: Keyword and search results in each database; Table S3: Excluded studies and reason; Table S4: Newcastle-Ottawa Scale for Observational trial.
