# Supplementary material for: Audiovestibular Dysfunction Related to Anti-Phospholipid Syndrome: A Systematic Review
Source: Diagnostics (Basel). 2024 Nov 11;14(22):2522. doi: 10.3390/diagnostics14222522 (PMC11592568; doi:10.3390/diagnostics14222522)
Supplement: Supplementary file 1 [file diagnostics-14-02522-s001.zip › diagnostics-3299629-supplementary.pdf]

List of content in supplement materials

Audiovestibular Dysfunction related to anti-phospholipid syndrome: A Systematic Review

Jiann-Jy Chen [1,2,†], Chih-Wei Hsu [3,†], Yen-Wen Chen [1,†], Tien-Yu Chen [4,5], Bing-Yan Zeng [6,7,*,‡] and Ping-Tao Tseng [1,6,8,9,*,‡]

[1] Prospect Clinic for Otorhinolaryngology & Neurology, Kaohsiung City 81166, Taiwan
[2] Department of Otorhinolaryngology, E-Da Cancer Hospital, I-Shou University, Kaohsiung 807066, Taiwan
[3] Department of Psychiatry, Kaohsiung Chang Gung Memorial Hospital and Chang Gung University College of Medicine, Kaohsiung, Taiwan
[4] Department of Psychiatry, Tri-Service General Hospital; School of Medicine, National Defense Medical Center, Taipei, Taiwan
[5] Institute of Brain Science, National Yang Ming Chiao Tung University, Taipei 112, Taiwan
[6] Institute of Biomedical Sciences, National Sun Yat-sen University, Kaohsiung, Taiwan
[7] Department of Internal Medicine, E-Da Dachang Hospital, I-Shou University, Kaohsiung, Taiwan
[8] Department of Psychology, College of Medical and Health Science, Asia University, Taichung, Taiwan
[9] Institute of Precision Medicine, National Sun Yat-sen University, Kaohsiung City, Taiwan
* Correspondence: holdinggreat@yahoo.com.tw (B.-Y.Z.); ducktseng@gmail.com (P.-T.T.)
† These authors contributed equally to this work.
‡ These authors contributed equally to this work.

Table S1	PRISMA 2020 checklist of current systematic review
Table S2	Keyword and search results in each database
Table S3	Excluded studies and reason
Table S4	Newcastle-Ottawa Scale for Observational trial

Table S1: PRISMA 2020 checklist of current systematic review

Section and Topic	Item #	Checklist item	Page where item is reported
**TITLE**			
Title	1	Identify the report as a systematic review.	1
**ABSTRACT**			
Abstract	2	See the PRISMA 2020 for Abstracts checklist.	3
**INTRODUCTION**			
Rationale	3	Describe the rationale for the review in the context of existing knowledge.	4-5
Objectives	4	Provide an explicit statement of the objective(s) or question(s) the review addresses.	4-5
**METHODS**			
Eligibility criteria	5	Specify the inclusion and exclusion criteria for the review and how studies were grouped for the syntheses.	6-7
Information sources	6	Specify all databases, registers, websites, organisations, reference lists and other sources searched or consulted to identify studies. Specify the date when each source was last searched or consulted.	6-7
Search strategy	7	Present the full search strategies for all databases, registers and websites, including any filters and limits used.	6-7
Selection process	8	Specify the methods used to decide whether a study met the inclusion criteria of the review, including how many reviewers screened each record and each report retrieved, whether they worked independently, and if applicable, details of automation tools used in the process.	6-7
Data collection process	9	Specify the methods used to collect data from reports, including how many reviewers collected data from each report, whether they worked independently, any processes for obtaining or confirming data from study investigators, and if applicable, details of automation tools used in the process.	6-7
Data items	10a	List and define all outcomes for which data were sought. Specify whether all results that were compatible with each outcome domain in each study were sought (e.g. for all measures, time points, analyses), and if not, the methods used to decide which results to collect.	6-7
	10b	List and define all other variables for which data were sought (e.g. participant and intervention characteristics, funding sources). Describe any assumptions made about any missing or unclear information.	6-7
Study risk of bias assessment	11	Specify the methods used to assess risk of bias in the included studies, including details of the tool(s) used, how many reviewers assessed each study and whether they worked independently, and if applicable, details of automation tools used in the process.	6-7
Effect measures	12	Specify for each outcome the effect measure(s) (e.g. risk ratio, mean difference) used in the synthesis or presentation of results.	6-7
Synthesis methods	13a	Describe the processes used to decide which studies were eligible for each synthesis (e.g. tabulating the study intervention characteristics and comparing against the planned groups for each synthesis (item #5)).	Not done
	13b	Describe any methods required to prepare the data for presentation or synthesis, such as handling of missing summary statistics, or data conversions.	Not done

Section and Topic	Item #	Checklist item	Page where item is reported
	13c	Describe any methods used to tabulate or visually display results of individual studies and syntheses.	Not done
	13d	Describe any methods used to synthesize results and provide a rationale for the choice(s). If meta-analysis was performed, describe the model(s), method(s) to identify the presence and extent of statistical heterogeneity, and software package(s) used.	Not done
	13e	Describe any methods used to explore possible causes of heterogeneity among study results (e.g. subgroup analysis, meta-regression).	Not done
	13f	Describe any sensitivity analyses conducted to assess robustness of the synthesized results.	Not done
Reporting bias assessment	14	Describe any methods used to assess risk of bias due to missing results in a synthesis (arising from reporting biases).	6-7
Certainty assessment	15	Describe any methods used to assess certainty (or confidence) in the body of evidence for an outcome.	6-7
**RESULTS**			
Study selection	16a	Describe the results of the search and selection process, from the number of records identified in the search to the number of studies included in the review, ideally using a flow diagram.	8-9
	16b	Cite studies that might appear to meet the inclusion criteria, but which were excluded, and explain why they were excluded.	8-9
Study characteristics	17	Cite each included study and present its characteristics.	8-9
Risk of bias in studies	18	Present assessments of risk of bias for each included study.	8-9
Results of individual studies	19	For all outcomes, present, for each study: (a) summary statistics for each group (where appropriate) and (b) an effect estimate and its precision (e.g. confidence/credible interval), ideally using structured tables or plots.	10-16
Results of syntheses	20a	For each synthesis, briefly summarise the characteristics and risk of bias among contributing studies.	Not done
	20b	Present results of all statistical syntheses conducted. If meta-analysis was done, present for each the summary estimate and its precision (e.g. confidence/credible interval) and measures of statistical heterogeneity. If comparing groups, describe the direction of the effect.	Not done
	20c	Present results of all investigations of possible causes of heterogeneity among study results.	Not done
	20d	Present results of all sensitivity analyses conducted to assess the robustness of the synthesized results.	Not done
Reporting biases	21	Present assessments of risk of bias due to missing results (arising from reporting biases) for each synthesis assessed.	10-19
Certainty of evidence	22	Present assessments of certainty (or confidence) in the body of evidence for each outcome assessed.	10-19
**DISCUSSION**			
Discussion	23a	Provide a general interpretation of the results in the context of other evidence.	20
	23b	Discuss any limitations of the evidence included in the review.	20
	23c	Discuss any limitations of the review processes used.	20

Section and Topic	Item #	Checklist item	Page where item is reported
	23d	Discuss implications of the results for practice, policy, and future research.	20
**OTHER INFORMATION**			
Registration and protocol	24a	Provide registration information for the review, including register name and registration number, or state that the review was not registered.	19
	24b	Indicate where the review protocol can be accessed, or state that a protocol was not prepared.	19
	24c	Describe and explain any amendments to information provided at registration or in the protocol.	19
Support	25	Describe sources of financial or non-financial support for the review, and the role of the funders or sponsors in the review.	21
Competing interests	26	Declare any competing interests of review authors.	21
Availability of data, code and other materials	27	Report which of the following are publicly available and where they can be found: template data collection forms; data extracted from included studies; data used for all analyses; analytic code; any other materials used in the review.	21

The current checklist followed the latest PRISMA 2020 guideline [1].

Table S2: Keyword and search results in each database

Database	Keyword	Filter	Date*	Result
PubMed	(anti-phospholipid syndrome OR anti-phospholipid antibody OR anti-cardiolipin OR lupus anticoagulant OR beta-2 glycoprotein) AND (hearing loss OR sensorineural hearing loss OR SNHL OR audiology OR tinnitus OR vertigo OR vestibular OR dizziness OR temporal bone study)	N/A	2024/6/20	118
Embase	(anti-phospholipid syndrome OR anti-phospholipid antibody OR anti-cardiolipin OR lupus anticoagulant OR beta-2 glycoprotein) AND (hearing loss OR sensorineural hearing loss OR SNHL OR audiology OR tinnitus OR vertigo OR vestibular OR dizziness OR temporal bone study)	N/A	2024/6/20	799
ClinicalKey	(anti-phospholipid syndrome OR anti-phospholipid antibody OR anti-cardiolipin OR lupus anticoagulant OR beta-2 glycoprotein) AND (hearing loss OR sensorineural hearing loss OR SNHL OR audiology OR tinnitus OR vertigo OR vestibular OR dizziness OR temporal bone study)	N/A	2024/6/20	804
Web of Science	(anti-phospholipid syndrome OR anti-phospholipid antibody OR anti-cardiolipin OR lupus anticoagulant OR beta-2 glycoprotein) AND (hearing loss OR sensorineural hearing loss OR SNHL OR audiology OR tinnitus OR vertigo OR vestibular OR dizziness OR temporal bone study)	N/A	2024/6/20	44
ScienceDirect on line	(anti-phospholipid syndrome) AND (hearing loss OR audiology OR vestibular OR temporal bone study)	N/A	2024/6/20	4050

*: initial search date on 2024/1/27, final update on 2024/6/20

Abbreviation: N/A: not applied

Table S3: Excluded studies and reason

Reason	Numbers	References
Review article	5	[2-6]
Mixed different autoimmune diseases but not specific to anti-phospholipid syndrome	2	[7,8]
Meta-analysis	1	[9]
Not related to anti-phospholipid syndrome	10	[10-19]
Not related to audiovestibular dysfunction	4	[20-23]

Table S4: Newcastle-Ottawa Scale for Observational trial

Study	Selection				Comparability	Exposure			Total
	Case definition	Representativeness	Control selection	Control definition	Comparability	Ascertainment	Same method	Non-Response rate	Summary
Williamson, K.A. (2021)[24]	*					*			2*
Erez, D. (2020)[25]	*	*				*			3*
Jeong, J. (2019)	*		*	*		*	*		5*
Mazzoccoli, C. (2019)[26]	*					*			2*
Ali, A.H. (2018)[27]	*					*			2*
Galicia-Lopez, A. (2016)[28]	*					*			2*
Passamonti, S.M. (2015)[29]	*		*	*		*	*		5*
Wang, J.G. (2009)[30]	*					*			2*
Suslu, N. (2009)[31]	*	*				*			3*

Gross, M. (2008)[32]	*	*			*		3*
Kang, K.T. (2008)[33]	*				*		2*
Cavallasca, J.A. (2007)[34]	*				*		2*
Bir, L.S. (2006)[35]	*				*		2*
Digiovanni, J.J. (2006)[36]	*				*		2*
Wiles, N.M. (2006)[37]	*	*			*		3*
Bachor, E. (2005)[38]	*	*	*	*	*	*	6*
Compadretti, G.C. (2005)[39]	*				*		2*
Marcucci, R. (2005)[40]	*		*	*	*	*	5*
Mouadeb, D.A. (2005)[41]	*	*			*		3*
Kremmer, S. (2004)[42]	*	*	*	*	*	*	6*

Toubi, E. (2004)[43]	*	*	*	*	*	5*
Lopez-Gentili, L.I. (2003)[44]	*				*	2*
Ruckenstein, M.J. (2002)[45]	*				*	2*
Hirose, K. (1999)[46]	*	*			*	3*
Toubi, E. (1997)[47]	*	*			*	3*
Hisashi, K. (1996)[48]	*				*	2*
Tumiati, B. (1995)[49]	*				*	2*
Vyse, T. (1994)[50]	*				*	2*
Hisashi, K. (1993)[51]	*				*	2*

* indicated this study have a good performance in this item

Reference list of supplement tables:

1. Page, M.J.; McKenzie, J.E.; Bossuyt, P.M.; Boutron, I.; Hoffmann, T.C.; Mulrow, C.D.; Shamseer, L.; Tetzlaff, J.M.; Akl, E.A.; Brennan, S.E.; et al. The PRISMA 2020 statement: an updated guideline for reporting systematic reviews. *Bmj* **2021**, *372*, n71, doi:10.1136/bmj.n71.
2. Agrup, C.; Luxon, L.M. Immune-mediated inner-ear disorders in neuro-otology. *Curr Opin Neurol* **2006**, *19*, 26-32, doi:10.1097/01.wco.0000194143.02171.46.
3. Brey, R.L.; Escalante, A. Neurological manifestations of antiphospholipid antibody syndrome. *Lupus* **1998**, *7 Suppl 2*, S67-74, doi:10.1177/096120339800700216.
4. Knight, J.S.; Branch, D.W.; Ortel, T.L. Antiphospholipid syndrome: advances in diagnosis, pathogenesis, and management. *Bmj* **2023**, *380*, e069717, doi:10.1136/bmj-2021-069717.
5. Riera, J.L.; Del, R.M.M.; Musuruana, J.L.; Cavallasca, J.A. Sudden Sensorineural Hearing Loss in Systemic Lupus Erythematosus and Antiphospholipid Syndrome: A Clinical Review. *Curr Rheumatol Rev* **2020**, *16*, 84-91, doi:10.2174/1573397115666191016101741.
6. Girasoli, L.; Cazzador, D.; Padoan, R.; Nardello, E.; Felicetti, M.; Zanoletti, E.; Schiavon, F.; Bovo, R. Update on Vertigo in Autoimmune Disorders, from Diagnosis to Treatment. *Journal of immunology research* **2018**, *2018*, 5072582, doi:10.1155/2018/5072582.
7. Hajas, A.; Szodoray, P.; Barath, S.; Sipka, S.; Rezes, S.; Zeher, M.; Sziklai, I.; Szegedi, G.; Bodolay, E. Sensorineural hearing loss in patients with mixed connective tissue disease: immunological markers and cytokine levels. *J Rheumatol* **2009**, *36*, 1930-1936, doi:10.3899/jrheum.081314.
8. Sharma, Y.K.; Chauhan, S. Overlap Syndrome with Rowell's Syndrome, Antiphospholipid Syndrome, Primary Sterility, and Sensorineural Hearing Loss: A Case Report, Brief Review, and Analysis of Cases of Rowell's Syndrome Reported from India and Abroad. *Indian J Dermatol* **2018**, *63*, 418-423, doi:10.4103/ijd.IJD_437_17.
9. Galli, M.; Luciani, D.; Bertolini, G.; Barbui, T. Lupus anticoagulants are stronger risk factors for thrombosis than anticardiolipin antibodies in the antiphospholipid syndrome: a systematic review of the literature. *Blood* **2003**, *101*, 1827-1832, doi:10.1182/blood-2002-02-0441.
10. Green, L.; Miller, E.B. Sudden sensorineural hearing loss as a first manifestation of systemic lupus erythematosus: association with anticardiolipin antibodies. *Clin Rheumatol* **2001**, *20*, 220-222, doi:10.1007/s100670170069.

11. Cho, C.H.; Jung, B.S.; Jung, J.H.; Lee, J.H.; Lee, J.H. Expression of autoantibodies in patients with sudden sensorineural hearing loss. *Ann Otol Rhinol Laryngol* **2013**, *122*, 131-134, doi:10.1177/000348941312200209.
12. Kobayashi, S.; Fujishiro, N.; Sugiyama, K. Systemic lupus erythematosus with sensorineural hearing loss and improvement after plasmapheresis using the double filtration method. *Internal medicine* **1992**, *31*, 778-781, doi:10.2169/internalmedicine.31.778.
13. Naarendorp, M.; Spiera, H. Sudden sensorineural hearing loss in patients with systemic lupus erythematosus or lupus-like syndromes and antiphospholipid antibodies. *J Rheumatol* **1998**, *25*, 589-592.
14. Li, X.; Chen, W.; Yi, H.; Xu, J.; Lu, M.; Guo, F.; Li, X.; Ye, J. The association between the levels of autoimmune related indicators and clinical condition in sudden deafness patients. *Ann Palliat Med* **2021**, *10*, 10535-10541, doi:10.21037/apm-21-2388.
15. Rajati, M.; Saghafi, M.; Rafatpanah, H.; Rasoulian, B.; Irani, S.; Soltankhah, M. Immunology-Rheumatology Approach to Sudden Sensorineural Hearing Loss. *Curr Rheumatol Rev* **2018**, *14*, 70-73, doi:10.2174/1573397112666161029221905.
16. Ballesteros, F.; Alobid, I.; Tassies, D.; Reverter, J.C.; Scharf, R.E.; Guilemany, J.M.; Bernal-Sprekelsen, M. Is there an overlap between sudden neurosensorial hearing loss and cardiovascular risk factors? *Audiol Neurootol* **2009**, *14*, 139-145, doi:10.1159/000171475.
17. Agarwal, K.; Thomas, N.; Taneja, V.; Beri, R.S.; Khanduri, U.; Puliyel, J.M. Plasma exchange in a child with systemic lupus erythematosus antiphospholipid antibodies and profound deafness. *Ann Trop Paediatr* **2002**, *22*, 109-110, doi:10.1179/atp.2002.22.1.109.
18. Agarwal, K.S.; Puliyel, J.M.; Khanduri, U. Sudden onset profound deafness in association with antiphospholipid antibodies in a child with SLE. *Indian Pediatr* **2000**, *37*, 1274-1276.
19. Paira, S.O. Sudden sensorineural hearing loss in patients with systemic lupus erythematosus or lupus-like syndrome and antiphospholipid antibodies. *J Rheumatol* **1998**, *25*, 2476-2477.
20. Carp, H.J.; Shoenfeld, Y. Anti-phospholipid antibodies and infertility. *Clin Rev Allergy Immunol* **2007**, *32*, 159-161, doi:10.1007/s12016-007-0010-2.
21. Yang, D.L.; Thomas, R.; Ford, A.F.; Cucchiara, B.L.; George, D.K.; Song, J.W. Vessel wall imaging in the diagnosis of antiphospholipid syndrome presenting as Moyamoya syndrome-A case report. *Neuroradiol J* **2024**, 19714009241247469, doi:10.1177/19714009241247469.

22. Nouduri, S.; Padmanabhan, R.; Hicks, R.; Abbott, M.A.; O'Brien, D.; Schlaug, G. Case report: MELAS and concomitant presumed antiphospholipid antibody syndrome in an adult woman. *Front Neurol* **2022**, *13*, 1043695, doi:10.3389/fneur.2022.1043695.
23. Lin, T.S.; Hsu, P.Y.; Chang, C.H.; Ko, C.L.; Kuo, Y.M.; Wu, Y.W.; Yen, R.F.; Wu, C.H.; Li, K.J.; Hsein, Y.C.; et al. Increased heterogeneity of brain perfusion is an early marker of central nervous system involvement in antiphospholipid antibody carriers. *PloS one* **2017**, *12*, e0182344, doi:10.1371/journal.pone.0182344.
24. Williamson, K.A.; Sanchez-Alvarez, C.; Duarte-Garcia, A. 28-Year-Old Man With Recurrent Vertigo, Syncope, and Progressive Memory Impairment. *Mayo Clin Proc* **2021**, *96*, 1309-1314, doi:10.1016/j.mayocp.2020.08.052.
25. Erez, D.; Abarbuch, E.; Natour, A.; Meyer, A.; Natour, A.; Dovrish, Z.; Levy, Y. Audiological disturbances in patients with primary antiphospholipid syndrome. *Lupus* **2020**, *29*, 533-538, doi:10.1177/0961203320904780.
26. Mazzoccoli, C.; Comitangelo, D.; D'Introno, A.; Mastropierro, V.; Sabba, C.; Perrone, A. Antiphospholipid syndrome: a case report with an unusual wide spectrum of clinical manifestations. *Auto Immun Highlights* **2019**, *10*, 9, doi:10.1186/s13317-019-0119-3.
27. Ali, A.H.; Salahuddin, Z.; Salim, R. Sudden sensorineural hearing loss: A missed opportunity for treatment. *Malays Fam Physician* **2018**, *13*, 29-30.
28. Galicia-Lopez, A.; Anda-Garay, J.C.; Garcia de la Pena, M. Bilateral sudden sensorineural hearing loss in a patient with microangiopathic antiphospholipid syndrome. *Reumatol Clin* **2016**, *12*, 175-177, doi:10.1016/j.reuma.2015.05.011.
29. Passamonti, S.M.; Di Berardino, F.; Bucciarelli, P.; Berto, V.; Artoni, A.; Gianniello, F.; Ambrosetti, U.; Cesarani, A.; Pappalardo, E.; Martinelli, I. Risk factors for idiopathic sudden sensorineural hearing loss and their association with clinical outcome. *Thrombosis research* **2015**, *135*, 508-512, doi:10.1016/j.thromres.2015.01.001.
30. Wang, J.G.; Xie, Q.B.; Yang, N.P.; Yin, G. Primary antiphospholipid antibody syndrome: a case with bilateral sudden sensorineural hearing loss. *Rheumatol Int* **2009**, *29*, 467-468, doi:10.1007/s00296-008-0696-3.
31. Suslu, N.; Yilmaz, T.; Gursel, B. Utility of anti-HSP 70, TNF-alpha, ESR, antinuclear antibody, and antiphospholipid antibodies in the diagnosis and treatment of sudden sensorineural hearing loss. *Laryngoscope* **2009**, *119*, 341-346, doi:10.1002/lary.20050.
32. Gross, M.; Eliashar, R.; Ben-Yaakov, A.; Ulmansky, R.; Elidan, J. Prevalence and clinical significance of anticardiolipin, anti-beta2-

glycoprotein-1, and anti-heat shock protein-70 autoantibodies in sudden sensorineural hearing loss. *Audiol Neurootol* **2008**, *13*, 231-238, doi:10.1159/000115432.
33. Kang, K.T.; Young, Y.H. Sudden sensorineural hearing loss in a patient with primary antiphospholipid syndrome. *J Laryngol Otol* **2008**, *122*, 204-206, doi:10.1017/S0022215107007736.
34. Cavallasca, J.A.; Nasswetter, G.G.; Gutierrez, D.M.; Bercellini, E.A.; Brodsky, A.L. Sudden sensorineural hearing loss as a manifestation of primary antiphospholipid syndrome. *Joint Bone Spine* **2007**, *74*, 403-404, doi:10.1016/j.jbspin.2006.11.008.
35. Bir, L.S.; Esmeli, F.O.; Ardic, F.N. Sudden sensorineural hearing loss in a patient with primary antiphospholipid syndrome. *J Thromb Thrombolysis* **2006**, *21*, 277, doi:10.1007/s11239-006-6156-6.
36. Digiovanni, J.J.; Nair, P. Spontaneous recovery of sudden sensorineural hearing loss: possible association with autoimmune disorders. *J Am Acad Audiol* **2006**, *17*, 498-505, doi:10.3766/jaaa.17.7.5.
37. Wiles, N.M.; Hunt, B.J.; Callanan, V.; Chevretton, E.B. Sudden sensorineural hearing loss and antiphospholipid syndrome. *Haematologica* **2006**, *91*, ECR46.
38. Bachor, E.; Kremmer, S.; Kreuzfelder, E.; Jahnke, K.; Seidahmadi, S. Antiphospholipid antibodies in patients with sensorineural hearing loss. *Eur Arch Otorhinolaryngol* **2005**, *262*, 622-626, doi:10.1007/s00405-004-0877-y.
39. Compadretti, G.C.; Brandolini, C.; Tasca, I. Sudden sensorineural hearing loss in lupus erythematosus associated with antiphospholipid syndrome: case report and review. *Ann Otol Rhinol Laryngol* **2005**, *114*, 214-218, doi:10.1177/000348940511400308.
40. Marcucci, R.; Alessandrello Liotta, A.; Cellai, A.P.; Rogolino, A.; Berloco, P.; Leprini, E.; Pagnini, P.; Abbate, R.; Prisco, D. Cardiovascular and thrombophilic risk factors for idiopathic sudden sensorineural hearing loss. *Journal of thrombosis and haemostasis : JTH* **2005**, *3*, 929-934, doi:10.1111/j.1538-7836.2005.01310.x.
41. Mouadeb, D.A.; Ruckenstein, M.J. Antiphospholipid inner ear syndrome. *Laryngoscope* **2005**, *115*, 879-883, doi:10.1097/01.MLG.0000158666.15447.37.
42. Kremmer, S.; Kreuzfelder, E.; Bachor, E.; Jahnke, K.; Selbach, J.M.; Seidahmadi, S. Coincidence of normal tension glaucoma, progressive sensorineural hearing loss, and elevated antiphosphatidylserine antibodies. *Br J Ophthalmol* **2004**, *88*, 1259-1262,

doi:10.1136/bjo.2003.040832.
43. Toubi, E.; Ben-David, J.; Kessel, A.; Halas, K.; Sabo, E.; Luntz, M. Immune-mediated disorders associated with idiopathic sudden sensorineural hearing loss. *Ann Otol Rhinol Laryngol* **2004**, *113*, 445-449, doi:10.1177/000348940411300605.
44. Lopez-Gentili, L.I.; Garate, G.; Riveros, D.; Ameriso, S.F. [Antiphospholipid antibodies in patients with vestibular manifestations. A study of 16 cases]. *Rev Neurol* **2003**, *36*, 112-117.
45. Ruckenstein, M.J.; Prasthoffer, A.; Bigelow, D.C.; Von Feldt, J.M.; Kolasinski, S.L. Immunologic and serologic testing in patients with Meniere's disease. *Otol Neurotol* **2002**, *23*, 517-520; discussion 520-511, doi:10.1097/00129492-200207000-00021.
46. Hirose, K.; Wener, M.H.; Duckert, L.G. Utility of laboratory testing in autoimmune inner ear disease. *Laryngoscope* **1999**, *109*, 1749-1754, doi:10.1097/00005537-199911000-00005.
47. Toubi, E.; Ben-David, J.; Kessel, A.; Podoshin, L.; Golan, T.D. Autoimmune aberration in sudden sensorineural hearing loss: association with anti-cardiolipin antibodies. *Lupus* **1997**, *6*, 540-542, doi:10.1177/096120339700600611.
48. Hisashi, K.; Komune, S.; Komiyama, S.; Nakamura, K. [Sudden sensorineural hearing loss associated with anticardiolipin antibody]. *Nihon Jibiinkoka Gakkai kaiho* **1996**, *99*, 1157-1161, doi:10.3950/jibiinkoka.99.1157.
49. Tumiati, B.; Casoli, P. Sudden sensorineural hearing loss and anticardiolipin antibody. *Am J Otolaryngol* **1995**, *16*, 220, doi:10.1016/0196-0709(95)90109-4.
50. Vyse, T.; Luxon, L.M.; Walport, M.J. Audiovestibular manifestations of the antiphospholipid syndrome. *J Laryngol Otol* **1994**, *108*, 57-59, doi:10.1017/s0022215100125848.
51. Hisashi, K.; Komune, S.; Taira, T.; Uemura, T.; Sadoshima, S.; Tsuda, H. Anticardiolipin antibody-induced sudden profound sensorineural hearing loss. *Am J Otolaryngol* **1993**, *14*, 275-277, doi:10.1016/0196-0709(93)90075-i.
